# Case report: Hashimoto’s thyroiditis after CD19 chimeric antigen receptor T-cell therapy

**DOI:** 10.3389/fimmu.2022.995496

**Published:** 2022-10-27

**Authors:** Panpan Chen, Yongming Xia, Wen Lei, Shuhan Zhong, Huawei Jiang, Lingling Ren, Wenbin Qian, Hui Liu

**Affiliations:** ^1^ Department of Hematology, The Second Affiliated Hospital, Zhejiang University School of Medicine, Hangzhou, China; ^2^ Department of Hematology, Yuyao People’s Hospital of Zhejiang Province, The Affiliated Yangming Hospital of Ningbo University, Ningbo, China

**Keywords:** thyroditis, CAR T, lyphoma, TgAb, irAEs

## Abstract

Chimeric antigen receptor (CAR)-T cell therapy is a novel cell therapeutic approach that is increasingly being used to treat patients with relapsed refractory B-cell lymphoma. Despite the efficacy of CAR T cell therapy, it has various adverse effects that can affect any organ in the body. The application of immune checkpoint inhibitors such as programmed death 1 (PD-1), programmed death ligand 1 (PDL-1), and cytotoxic T-lymphocyte antigen 4 (CTLA-4) antibodies has previously been reported to be associated with immune-related adverse events such as thyroid dysfunction and thyroiditis. Reports of immune-related adverse reactions after CAR T therapy are currently extremely rare, with only one case of a cytokine storm (CRS) combined with severe arthritis in a patient with ALL after treatment. Here, we describe two cases of Hashimoto’s thyroiditis secondary to CAR T therapy. Two patients with relapsed refractory diffuse large B-cell lymphoma developed elevated peroxidase and globulin antibodies secondary to CAR-T cell therapy and developed Hashimoto’s thyroiditis. Complete remission was achieved in two patients at 1 and 3 months after CAR-T cell therapy. The inflammation of the thyroid tissue may be directly or indirectly related to CAR T cell therapy, and the mechanisms needs to be further investigated.

## Case presentation

### Case 1

**Figure 1:**
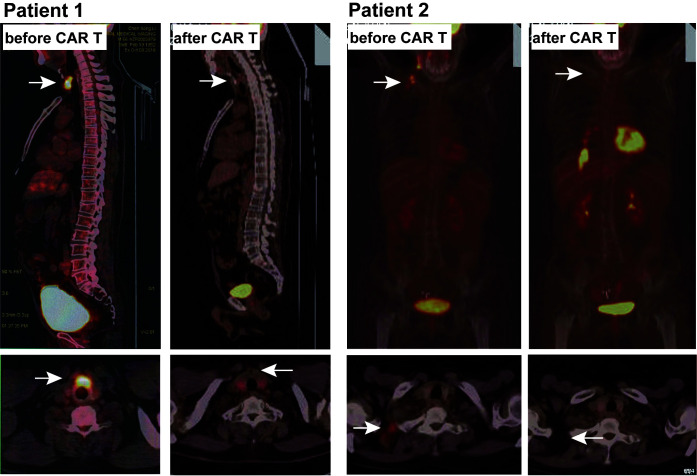
PET-CT images of two patients before and after CART treatment.

**Figure 2:**
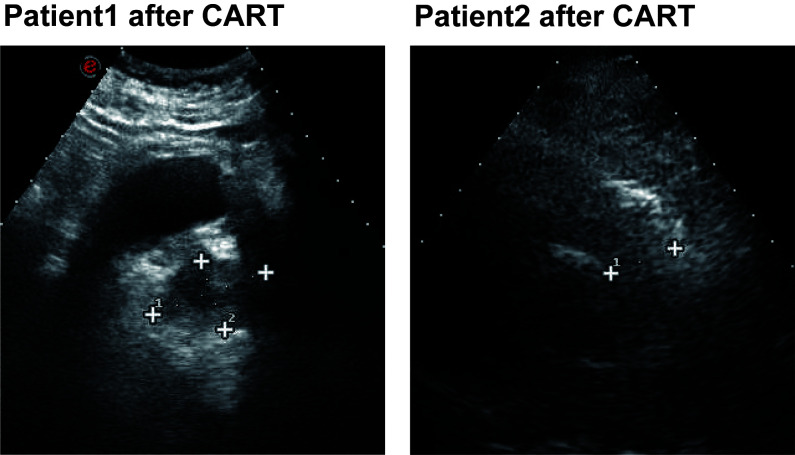
Ultrasound images of the thyroid gland after CART treatment in two patients.

A 65-year-old male presented with dull pain and mass on the left side of the neck without significant fever or chills, no nausea or vomiting, no abdominal pain, bloating or diarrhea, and no hoarseness. thyroid puncture was performed in 2017 and pathology suggested diffuse large b-cell lymphoma, GCB type, high-grade B-cell lymphoma, immunohistochemistry: CK-, EMA-, CD20+, CD79a+, CD3 little +, CD5 little +, CyclinD1-, BCL2-, BCL6 70%+, CD10 focal weak +, MUM1-, P53-, Kappa-, Lambda-, Ki-67 60%, CD19+, PD-L1-, FISH: MYC-, BCL2-, BCL6-. six times standard therapy with rituximab plus cyclophosphamide, doxorubicin, vincristine, and prednisone (R-CHOP), did not achieve partial remission (PR). Given rituximab plus gemcitabine, cisplatin and dexamethasone (R-GDP), the patient developed tinnitus in the right ear, so changed to rituximab plus gemcitabine oxaliplatin (R-Gemox) regimen chemotherapy. After four times of R-Gemox regimen chemotherapy, the patient performed autologous hemopoietic stem cell transplantation (HSCT) in April 2018. Subsequent PET-CT (**Figure 1**) suggested a hypodense thyroid nodule 1.8*2.7*3.4 cm^3^ with unclear borders, localized at the anterior borders above the lesion invading the anterior vocal cord union, SUVmax 16.38. The patient was proposed for CAR T therapy, peripheral lymphocytes were collected (**Table 1**). On November 8, 2018 fludarabine and cyclophosphamide (FC) pretreatment regimen was performed to clearance of lymphocytes, and CAR T cells were infused back on November 12 (**Table 2**). The disease was evaluated in partial remission at one month and complete remission at three months (**Table 3**). Ultrasound suggested thyroid nodule of 0.8*0.6 cm (**Figure 2**), thyroid ultrasound suggested thyroid inflammation, thyroid function T3, T4, TSH within normal range, anti-thyroid peroxidase antibody and thyroglobulin antibody were elevated (**Table 1**). Consider secondary Hashimoto’s thyroiditis.

**Table 1:** Summary of the 2 cases with thyroid irAEs after CAR T cell therapy.

Case summaries		Following pembrolizumab therapy						Overview of 18 FDG-PET/CT imaging			
Patient	thyroiditis-related irAE	TSH	T3	FT3	T4	ATG Ab	TPO Ab	TG	Baseline	After abnormal TFTs	Time from CAR T infusion, mo
Patient 1	Thyroiditis, transient	2.06	1.57	4.32	94.5	171.8	31.48	NA	Increased	Increased	3
Patient 2	Thyroiditis, transient	–	1.74	5.58	125.6	13.29	33.83	6.02	No increase	Increased	3

NA, not available.

TSH, 0.35 to 4.94 mIU/L.

Total T3, 0.98 to 2.33 nmol/L.

FT3, 2.43 to 6.01 pmol/L.

Total T4, 58.1 to 140.6 nmol/L.

TPO Ab, <5.61 IU/mL.

ATG Ab, <4.11 IU/mL.

TG, 3.5-77.0 μg/L.

**Table 2:** Dynamic changes of serum cytokines after CAR T cell therapy.

Time from CAR T infusion, days	Patient 1			Patient 2		
	IL-6	TNF-α	INF-γ	IL-6	TNF-α	INF-γ
D1	3.84	1.14	9.51	14.22	8.57	0.1
D4	5.05	3.55	5.52	130.35	80.48	0.1
D8	10.33	2.32	12.28	8.98	18.73	0.1
D11	4.63	1.8	13.39			
D30	22.61	0.1	0.1	3.11	0.19	3.93
D90	1.82	0.95	2.56	1.67	0.87	0.1
D180	3.33	0.46	1.13	11.94	2.91	6

IL-6, Interleukin-6; TNF-α, tumor necrosis factor-α; INF-γ, Interferon-γ.

IL-6, 0.0 to 16.6 pg/ml.

TNF-α, 0.0 to 5.2 pg/ml.

INF-γ, 0.0 to 17.3 pg/ml.

**Table 3:** Timeline of Patient 1.

Day	Event
2017/5/10	Diagnosis as diffuse large b-cell lymphoma
2017/5/10	R-CHOP
2017/6/13	R-CHOP
2017/7/4	R-CHOP
2017/7/25	R-CHOP
2017/8/15	R-CHOP
2017/9/5	R-CHOP
2017/10/17	R-GDP
2017/11/29	R-GeMox
2017/12/22	R-GeMox
2018/1/15	R-GeMox
2018/2/9	R-GeMox
2018/3/1	R-GeMox
2018/4/2	autologous hemopoietic stem cell transplantation (HSCT)
2018/10/29	peripheral lymphocytes collection
2018/11/8	fludarabine and cyclophosphamide (FC) pretreatment
2018/11/12	CAR T cells infusion
2018/12/14	partial remission
2019/02/20	complete remission

### Case 2

A 52-year-old female presented with right cervical lymph node enlargement without tenderness. A lymphoproliferative lesion was found during a neck biopsy at an outside hospital in October 2016. On reexamination 2 months later, the lymph node was enlarged by approximately 6* 8 cm without tenderness, with limited mobility, chest tightness, shortness of breath, no fever or chills, and no cough or sputum. A right cervical lymph node biopsy in December 2016 suggested (cervical lymph node) non-Hodgkin lymphoma, consistent with diffuse large b-cell lymphoma of germinal center origin, immunohistochemistry: CD3+, CD20+, Ki-67 60%+, CD79a+, CD30 partial+, CD5-, CD10+, BCL2+, BCL6 scattered+, MUM1-, PAX5+, CD21-, CyclinD1-, C-MYC-. ALK-, EBER-, CD15-. After the diagnosis was confirmed one course of chemotherapy with R-CHOP regimen, 3 courses of rituximab plus etoposide, cyclophosphamide, adriamycin, vincristine, prednisone (R-ECHOP) regimen, and 4 courses of R-CHOP regimen with partial remission assessed for efficacy. Lymph node enlargement 2 months after the end of chemotherapy and another lymph node biopsy in August 2018 suggested diffuse large b-cell lymphoma of germinal center origin and disease progression was considered. PET-CT (**Figure 1**) suggested lymphoma after chemotherapy: deep right sternocleidomastoid muscle and cervical sheath, right cervical root, left cervical sheath, bilateral supra- and inferior clavicular regions, mediastinal thoracic inlet and upper mediastinal vascular space, multiple deep axillary nodes of variable size on both sides Lymph nodes with varying degrees of increased FDG metabolism. Peripheral blood lymphocytes were collected on September 19, 2018. Pretreatment clearance of lympohcytes with FC protocol was performed on September 29, 2018 and CAR - t cell infusion was performed on October 4. One month later the disease was assessed to be in complete remission (**Table 4**). During follow-up, the patient had elevated TGAb, TMAb and TPOAb, but normal thyroid function (**Table 2**). Secondary Hashimoto’s thyroiditis was considered.

**Table 4:** Timeline of patient 2.

Day	Event
2017/1/5	Diagnosis as diffuse large b-cell lymphoma
2017/1/5	R-CHOP
2017/2/6	R-ECHOP
2017/3/3	R-ECHOP
2017/3/30	R-ECHOP
2017/4/28	R-CHOP
2017/5/19	R-CHOP
2017/6/13	R-CHOP
2017/7/4	R-CHOP
2018/9/19	peripheral lymphocytes collection
2018/9/29	fludarabine and cyclophosphamide (FC) pretreatment
2018/10/4	CAR T cells infusion
2018/11/6	complete remission
2019/01/9	complete remission

## Discussion

CAR - T cell therapy is a novel biological therapy in which T cells are genetically transduced to express specific CAR - T proteins that specifically recognize target antigens and kill target tumor cells (1). The major adverse effects of CAR - T cell therapy are cytokine release syndrome, neurotoxicity, and targeted and untargeted killing of healthy cells (2, 3). Li et al. reported a rare case of a patient with acute lymphoblastic leukemia who developed CRS with major arthritis after CAR T treatment and relief of joint symptoms after glucocorticoid therapy (4). No other immune-related adverse reactions (irAEs) were reported after CAR T cell therapy. Here, we report for the first time the progression of Hashimoto’s thyroiditis (HT) in two patients with diffuse large B-cell lymphoma treated with CAR T cells.

Both patients had diffuse large B cell GCB subtype and elevated thyroid-associated antibodies after CAR - T treatment, but thyroid function remained within normal limits. Thyroid ultrasound suggested localized thyroid inflammation. We believe that these two patients developed complications of Hashimoto’s thyroiditis (HT) after CAR - T treatment.

HT is a common autoimmune disease of the thyroid gland characterized by an enlarged or nodular thyroid gland with clinical manifestations of hyperthyroidism or hypothyroidism (5). In the pathophysiology of HT, B cells that produce inflammatory antibodies such as TGAb, TMAb, and TPOAb play a major role in thyroid tissue injury and are also involved in the infiltration of other lymphocytes, including plasma cells, natural killer cells, and macrophages (5). Several studies have shown that HT is an organ-specific autoimmune disease mediated by T cells (5). The relatively active function of helper T lymphocytes leads to decreased or even defective T lymphocyte function, resulting in a disruption of the balance between Th and T cells, leading to thyroid immune dysfunction (5). Several studies have reported the occurrence of HT after the use of immune checkpoint inhibitors (6). We report two cases of diffuse large B-cell lymphoma with HT after CAR T cell therapy. Ultimately, neither patient developed hypothyroidism and thyroid antibodies decreased at late follow-up. This is the first international report of HT complications after CAR T cell therapy.

In the previous literature, the use of immune checkpoint inhibitors has been reported to cause autoimmune associated inflammation in one organ or multiple systems throughout the body (7). Most immune responses occur in endocrine organs, particularly the thyroid (8). Related studies have shown that hypothyroidism occurs in 6-13% of patients receiving immunosuppressive therapy and hyperthyroidism in 3-16% (8). Adverse thyroid reactions occur over a wide range of time, from 1 day to 3 months after treatment, and even after cessation of treatment. More patients with thyroiditis present with only elevated antibodies and normal thyroid function.

The mechanisms underlying immunotherapy for hypothyroidism is not fully understood. There may be thyroid inflammation, in which both T cells and natural killer cells may be involved (7, 9). The mechanism of thyroid inflammation after CAR T therapy have not been investigated. In conclusion, thyroid inflammation is associated with a combination of immunopathogenesis, with the entry of CAR - T cells into the bloodstream, the balance of T cells in the thyroid is disrupted, the role of T helper lymphocytes increases and the function of regulatory T cells decreases, which eventually leads to the production of antibodies by B lymphocytes and cytotoxic effects by antibody-dependent thyroid cells. On the other hand, when CAR - T cells enter the human body, cytotoxic CAR - T cells upregulate the expression of Fas and FasL in thyroid cells by releasing a large number of cytokines, thus promoting apoptosis and eventually leading to HT. the above are only speculations on the possible mechanisms, and the specific mechanisms need to be further investigated.

## Conclusion

We reported for the first time the development of HT in two patients with diffuse large B-cell lymphoma treated with CAR T cells; these two patients presented only show elevated thyroid antibodies and no thyroid dysfunction was found, requiring no treatment.

## Data availability statement

The original contributions presented in the study are included in the article/supplementary material. Further inquiries can be directed to the corresponding author.

## Ethics statement

Written informed consent was not obtained from the individual(s) for the publication of any potentially identifiable images or data included in this article.

## Author contributions

PC and YX performed the literature. WL performed CART cell detection after treatment. HL and SZ collected the data. WQ and HL revised the work. HJ and LR supported the study and reviewed the manuscript. All authors contributed to the article and approved the submitted version.

## Conflict of interest

The authors declare that the research was conducted in the absence of any commercial or financial relationships that could be construed as a potential conflict of interest.

## Publisher’s note

All claims expressed in this article are solely those of the authors and do not necessarily represent those of their affiliated organizations, or those of the publisher, the editors and the reviewers. Any product that may be evaluated in this article, or claim that may be made by its manufacturer, is not guaranteed or endorsed by the publisher.
